# The genesis of the AIDS policy and AIDS Space in Brazil (1981-1989)

**DOI:** 10.1590/S1518-8787.2016050005801

**Published:** 2016-07-12

**Authors:** Sandra Garrido de Barros, Ligia Maria Vieira-da-Silva

**Affiliations:** I Programa Integrado de Pesquisa em Política, Gestão e Avaliação. Instituto de Saúde Coletiva. Universidade Federal da Bahia. Salvador, BA, Brasil; II Programa de Pós-Graduação em Saúde Coletiva. Instituto de Saúde Coletiva. Universidade Federal da Bahia. Salvador, BA, Brasil

**Keywords:** Acquired Immunodeficiency Syndrome history, Policy Making, Sociology, Medical, Social Determinants of Health, Equity in Health, Bourdieu

## Abstract

**OBJECTIVE:**

To analyze the genesis of the policy for controlling AIDS in Brazil.

**METHODS:**

Socio-historical study (1981-1989), based on Bordieu’s genetic sociology, by document analysis, bibliographical review, and in-depth interviews. It consisted of a connection between the analysis of the paths of 33 agents involved in the creation of a social space focusing on AIDS-related issues and the historical possibility conditions of the drafting of a specific policy.

**RESULTS:**

AIDS Space is a gathering point for the paths of agents from several social fields (medical, scientific, political, and bureaucratic fields). A specific space for relationships, which enabled the drafting of a policy for controlling the AIDS epidemic, but also a place where the authority to talk about the meaning of the disease, the methods to prevent and treat it was under dispute. The analysis showed how the various structures (democratic administrations in Sao Paulo and at the national level, with public health officers taking important positions) and the lack of a specific therapy contributed to social agents of different ranks and backgrounds to initially set prevention as a priority.

**CONCLUSIONS:**

The rise of the sanitary movement, the organization of SUS, and the dominance of the medical field at the AIDS Space contributed to foster treatment as a part of the measures to control the epidemic. These conditions allowed drafting a policy based on the integrality of care, by linking prevention and treatment in the following decade, with important participation from state bureaucracy and researchers.

## RESUMO

**OBJETIVO:**

Analisar a gênese da política de controle da aids no Brasil.

**MÉTODOS:**

Estudo sócio-histórico (1981-1989), orientado pela sociologia genética de Bourdieu, por meio de análise documental, revisão bibliográfica e entrevistas em profundidade. Consistiu na articulação entre a análise das trajetórias de 33 agentes envolvidos na criação de um espaço social voltado para as questões relativas à aids e as condições históricas de possibilidade para a formulação de uma política específica.

**RESULTADOS:**

O Espaço Aids constituiu-se como produto do encontro da trajetória de agentes de diversos campos sociais (médico, científico, político e burocrático). Um espaço específico de relações, que possibilitou a formulação de uma política para o controle da epidemia da aids, mas onde também estava em disputa a autoridade de falar sobre o significado da doença, suas formas de prevenção e tratamento. A análise mostrou como as estruturas (governos democráticos no estado de São Paulo e no âmbito nacional, com sanitaristas assumindo posições importantes) e a ausência de terapia específica contribuíram para que agentes sociais com disposições e formações diversas formulassem uma política que priorizou inicialmente a prevenção.

**CONCLUSÕES:**

A ascensão do movimento sanitário, a constituição do SUS e a dominância do campo médico no Espaço Aids contribuíram para a valorização do tratamento, como parte das medidas de controle da epidemia. Essas condições possibilitaram a formulação de uma política baseada na integralidade das ações, articulando prevenção e tratamento, na década seguinte, com importante participação da burocracia estatal e de pesquisadores.

## INTRODUCTION

To analyze the genesis of a public health care policy is to seek to understand the historical and social circumstances that enabled the State to intervene to implement specific measures for controlling a certain health problem^22^.

The studies on the origins of Brazil’s AIDS policy, which were reviewed, focused on analyses of state cases, especially Sao Paulo^a,b,c,d^or on the relationships between non-government organizations (NGO) and the State^11,e,f,g^, whether by highlighting the political focus of these organizations or by emphasizing the drafting and implementation of the policy for specific groups^10^ at the different levels of the health care system^h,i^. Those who analyzed the government response to the epidemic at the national level have not investigated the participation of medical and scientific fields, nor have they considered the social and professional paths of agents^13,15,18^.

By putting the concept of social space developed by Bourdieu^5^ into practice, Pinell et al.^22^ studied the universe of possibilities for the rise of a movement to fight AIDS in France, its structure, and its dynamics of relationships of AIDS Space agents between 1981 and 1996. This space was created in that country through a popular mobilization that aimed to replace state institutions in the performance of this public role. The policy against AIDS was drafted by the initiatives of militant associations, the advancements in the medical field, and the illness social representation as a threat to French society.

The study by Mendonça et al.^17^, based on Bordieu’s field concept, connected the position at the “HIV/AIDS field” with entrepreneurial action methods. Their analysis focused on the medical field and on specific associations, but did not explore the interaction between the paths of agents and conditions of possibility. The development of a space for fighting AIDS is understood to depend on the medical knowledge on the disease, but not to fulfill all the requirements of a field, as proposed by Bordieu. The concept of AIDS Space as suggested by Pinell et al.^22^ is more appropriate because it is a space of relationships among the agents in different social fields.

The studies reviewed do not shed light on how the AIDS Space was organized in Brazil (composition, agents, disputes, interests) nor do they explain why a policy was drafted at a time when there was no evidence on its extent and vulnerability. Neither have they systematically analyzed the liaisons between agents, the points of view or strategies they chose regarding the policy on AIDS, concerning their fields of origin.

This study on the genesis of Brazil’s AIDS policy provides elements that help fill these gaps, based on Bourdieu’s reflexive sociology^3-8^.

## METHODS

A socio-historical study was conducted between 1981 and 1989, the time when the AIDS policy had its genesis. This period was defined according to a broader research periodization comprising the interval between 1981 and 2001^j^, considering the main groups affected, times, and priority actions of the government response and its relationship with medical knowledge (Table 1).

**Table 1:** Times of the national policy on AIDS control, main groups affected, priority initiatives and their relationship with medical knowledge, 1981-2001.

Period	Main groups affected^a^	Medical knowledge	Policy on AIDS	Priority initiatives
1981-1984	Homosexuals	Discovery of the virus Blood test	The federal government does not have a specific policy	State initiatives
1985-1989	Homosexuals, people with hemophilia, and other recipients of blood and blood products	AZT	Creation of a national policy	Health surveillance and education initiatives
1990-1996	Injection drug users, heterosexuals	Conduction of trials for vaccine studies in Brazil Combined therapy (disease control, increased survival)	Consolidation of the national policy	Distribution of medications, 1^st^ loan agreement, funding of NGO
1997-2001	Feminization^b^ Aging Interiorization Impoverishment Increased survival	New medications (protease inhibitors), reduced collateral effects, disease control	Apex of the national policy: reduction of morbidity and mortality indicators (stabilized epidemic) and international recognition	Sustainability of the universal access strategy

AZT: azithromycin; NGO: non-government organizations

^a^ Source: Epidemiological AIDS Bulletins, Ministry of Health.

^b^ The ratio between genders between 1980 and 1990 was 6.5:1, and in the period between 1991 and 2001 it was 2.4:1.

The concept of social space is based on the idea of difference: agents are distributed in it according to the various kinds of capital^5^ (Table 2). In turn, the concept of field corresponds to a network of relationships, a relatively autonomous microcosm comprising agents and institutions that have *habitus*, common perception and action schemes, and *illusio*, a shared interest^4,7^. The AIDS Space was operationalized as a space of relationships between agents in several fields, with common interest in a problem: AIDS^22^. To rebuild the dynamics of this space and its connections with the medical field, the Collective Health Space^25,k^ and the political field, we analyzed the social and professional trajectories of 33 agents (in-depth interviews) (Table 3) and the historical conditions for the rise of the policy (document and bibliographical sources).

**Table 2:** Definition of the different kinds of capital.

Type of capital	Description	Source
Cultural	Set of assets related to incorporated knowledge (being competent in a knowledge domain, being cultured, have a good mastery on language), owning cultural assets (books, dictionaries, instruments, machines) and, in its institutionalized state, to degrees, diplomas, and being approved in professional admission tests, that is, to the recognition of skills by the State.	Bourdieu^7^ (2008)
Social	Capital of relationships, regarding the gains associated with the existence of a network of real or potential connections, more or less institutionalized, of belonging to a group.	Bourdieu^3^ (1980)
Symbolic	Transmutation of the various species of capital into recognition capital by the agents in the social space	Bourdieu^5^ (1996)
Political	Related to the mobilization ability of an agent, it is a species of personally-obtained social and symbolic capital, which results from personal notoriety and popularity capital (being known and recognized), or by the delegation of an organization that holds this kind of capital, such as parties or sindicates. It can be achieved through the access to traditional political positions (positions in a party, in branches of power, in the network of companies related to parties, or by taking elective offices).	Bourdieu^6^ (2001) Matonti and Poupeau^16^ (2004)
Militant	Set of knowledge and practices implemented in collective initiatives and struggles between or within parties; it is incorporated through techniques and dispositions to act, intervene, or simply obey. Under certain conditions, it can be a path to political capital when, for example, an agent is associated with the personification and disclosure of an initiative that allows them to convert their acquired militant notoriety into more institutionalized political investments, such as political parties.	Matonti and Poupeau^16^ (2004) Garcia^12^ (2005)
Bureaucratic	A capital that has power over other kinds of capital, it also corresponds to the power of nomination, controlling information and standardization, and also defining and imposing legitimate state categories.	Bourdieu^8^ (2012)

**Table 3:** Profile of the subjects interviewed, according to college diplomas, year when joined AIDS Space, insertion sub-space, volume of scientific, bureaucratic, political, and militant capital, relationship with AIDS, and presence at AIDS Space during the rise of the national policy for controlling the epidemic, 1985.

E	Undergraduate diploma (institution, year)	Year when joined the space	Sub-space	Volume of capital (1983-1986)					Relationship with AIDS	Rise of PNAids (1985)

				Cultural	Scientific	Bureaucratic	Political	Militant		
1	Social sciences (PUC-SP, 1992)	1983	Bureaucratic	HC	-	-	-	-	Professional	X
2	Medicine (UFBA)	1993		-	-	-	-	-	Professional	-
3	Law (PUC-SP, N/I)	1983	Militant	CHE	-	-	B	B	Professional/ Personal	X
4	Medicine (UFBA, 1969)	1983	Scientific	G	AA	B	B	-	Professional	X
5	Social sciences (UFRJ, 1988)	1989	Militant	-	-	-	-	-	Professional	-
6	Philosophy (Unesp, N/I)	1994	Militant	-	-	-	-	-	Personal HIV+	-
7	Medicine (UFRJ, 197?)	1983	Scientific	CHE	-	-	-	-	Professional	-
8	Social Psychology (US, 1968)	1983	Militant	G	M	-	-	B	Political	X
9	Medicine (UFBA, 1965)	1985	Scientific	G	A	-	-	-	Research	X
10	Medicine (UFBA, 1968)	1985	Bureaucratic	G	B	AA	B	-	Professional	X
11	Medicine (UFPI, 1983)	1986	Bureaucratic	CHE	-	A	-	-	Professional/ Personal	X
12	Business Administration (Methodist University Center – SP, after 2001)	1986	Militant	-	-	-	-	-	Personal HIV+	-
13	Hospitality (Renascença Hebrew School, 1994)	1994	Militant	-	-	-	-	-	Personal HIV+	-
14	Business Administration (UCSal, after 2001)	1987	Militant	-	-	-	-		-	-
15	Tourism (N/I)	1986	Bureaucratic	CHE	-	B	-	-	Professional	-
16	Medicine (EBMSP, 1981)	1990	Scientific	-	-	-	-	-	Professional	-
17	Sociology (Sao Paulo School of Political Sociology, N/I)	1992	Bureaucratic	-	-	-	-	-	Professional	-
18	Mathematics (UBA, 1972)	1986	Militant	G	M	-	B	A	Personal HIV+	-
19	Medicine (USP, 1961)	1983	Scientific	G	A	A	B	-	Professional	X
20	Social Sciences (USP, 1968)	1983	Militant	G	M	-	B	A	Political	X
21	Economics (UnB, after 2001)	1986	Bureaucratic	ES	-	-	-	-	Professional	-
22	Psychology (UFBA, 1991)	1987	Militant	-	-	-	-	-	Personal	-
23	Medicine (UFBA, 1989)	1990	Bureaucratic	-	-	-	-	-	-	-
24	Medicine (EMESCAM, 1975)*	1985	Bureaucratic	CHE	-	A	-	B	Professional	X
25	Social Sciences (Unesp, 1987)	1988	Scientific	-	-	-	-	-	-	-
26	Medicine (Unesp, 1973)	1983	Bureaucratic	CHE	-	A	-	B	Professional	X
27	Medicine (UFRJ, 1986)	1986	Bureaucratic	CHE	-	A	-	-	Professional	-
28	Data processing technologist (N/I)	1993	Bureaucratic	-	-	-	-	-	Professional	-
29	Psychology (PUC-RJ, 1978)	1986	Militant	G	B	-	B	A	Professional	-
30	Medicine (UERJ, 1985)	1986	Scientific	CHE	-	-	-	-	Professional	-
31	Medicine (Unifesp, 1973)	1983	Scientific	G	M	-	-	-	Professional	X
32	Psychology (USP, 1977)	1984	Scientific	-	-	-	-	-	Professional	X
33	Psychology (UERJ, 1985)	1985	Militant	G	P	-	P	A	Political	X

E: subject interviewed; PUC-SP: Pontifícia Universidade Católica de São Paulo; UFBA: Universidade Federal da Bahia; N/I: no information; UFRJ: Universidade Federal do Rio de Janeiro; Unesp: Universidade Estadual Paulista “Júlio de Mesquita Filho”; US: University of Sussex; UFPI: Universidade Federal do Piauí; UCSal: Universidade Católica de Salvador; EBMSP: Escola Bahiana de Medicina e Saúde Pública; UBA: Universidad de Buenos Aires; USP: Universidade de São Paulo; UnB: Universidade de Brasília; EMESCAM: Escola Superior de Ciências da Santa Casa de Misericórdia de Vitória; PUC-RJ: Pontifícia Universidade Católica do Rio de Janeiro; UERJ: Universidade do Estado do Rio de Janeiro; UNIFESP: Universidade Federal de São Paulo; HC: high school; CHE: complete higher education; G: graduate; ES: elementary school; AA: very high scientific capital volume; M: medium scientific capital volume; A: high scientific capital volume; B: low scientific capital volume; PNAids: Brazil’s National Policy on AIDS

* E-mail contact

The sample was delimited through the saturation of oral information, which were confirmed and complemented by document analysis. The classification of the agents according to their social fields was conducted according to volume indicators of scientific, bureaucratic, militant, and political capital, as proposed by Vieira-da-Silva and Pinell^25^ (Table 4).

**Table 4:** Criteria for analyzing the composition of different species of capital during the rise of AIDS Space in Brazil, 1981-1986a.

Volume of capital	Very high (HH)	High (H)	Medium (M)	Low (L)
Type of capital				
Scientific				
	International recognition	National recognition	Local recognition	Master’s degree
	International awards	Full professor	Physican’s degree	-
	Coordinating research or structuring projects funded by international agencies	Coordinating projects funded by national agencies	Taking part in research projects related to the response to the epidemic	-
Bureaucratic^b^				
Occupying technical positions	Management positions in international agencies (UNAids, PAHO, WHO) Managers in the National STD/AIDS Program or higher positions with power of interfering in the AIDS policy	Management of state programs Technicians in the National STD/AIDS Program Participation in meetings for defining the MH’s policy	Intermediate management positions in municipal health care offices (municipal programs) Technicians in state programs	Technical positions (advisor, consultant)
Political				
Occupying administrative positions that result in specifically political liaison	Health minister, agency presidents	State health secretary	Municipal health secretary	Other politically-appointed technical positions
Participation in political parties	National manager	State manager of a political party	Municipal manager of political parties	Member of a political party
Participation in elective offices	Senator, congressman	Assemblyman	Alderman	-
Militant				
Professional and popular leaders	A charismatic leader who is capable of mobilizing and gathering people (high symbolic capital)	NGO or social movement manager	Intermediate NGO or social movement staff	NGO or social movement militants

UNAids: Joint United Nations Program on HIV/AIDS; PAHO: Pan American Health Organization; WHO: World Health Organization; MH: Ministry of Health; NGO: non-government organization

^a^ Adapted from Vieira-da-Silva and Pinell^22^.

^b^ Measured based on occupied positions, whereas the technical office occupation requires titles (acquired cultural capital) suitable for the position in question.

The project was approved by the Universidade Federal da Bahia’s Collective Health Institute’s Research Ethics Committee. The interviewed subjects signed informed consent forms and agreed to have their names disclosed.

## RESULTS AND DISCUSSION

### The Construction of the AIDS Space (1981-1984)

The epidemics arrived in Brazil in the early 1980s, when AIDS was not widely known and no efficient therapeutic response existed. From a political point of view, it was a period marked by the transition to democracy after the military dictatorship that started in 1964.

The first news reports about the disease were treated as a foreign problem by the media^2^ and contributed to it being seen as a gay disease, the so-called “gay plague” ^l^. The first cases, which were concentrated in Sao Paulo, were identified between 1982 and 1983, by dermatologist Valéria Petri. The patients had Kaposi’s sarcoma, which is very unusual in young people. No blood tests existed; diagnose was clinical and therefore questioned by some physicans, according to the interviewed testimonial, “[...] some colleagues insisted I was lying” (E31, 6/20/2011). Despite the role of the press in giving the first reports on AIDS, only in 1983 were the first Brazilian cases reported^1,2^.

Despite first denying the disease, the gay movement was fundamental to recognize AIDS as a problem that required specific measures. The questioning stance on medical field was a result from the historical relationship of some physicians with homosexuality, who viewed it as a mental illness.

“[...] Militants tended to think that it was another lie from physicans [...]. Yet another way to control homosexuality, to get people to quit these behaviors” (E8, 5/16/2011).

Afterwards, this group started reinforcing medical discourse, probably as a result from the exchange with foreign groups who brought reports of fear and death, and also because of the threat to the developing gay trade^14,21,m^.

“[...] I spent a week in New York*.* [...] I heard about people dying like flies. [...] almost no one believed it” (E8, 5/16/2011).

The epidemic transformed the gay rights movement, by causing its sexual freedom proposals to lose momentum and reducing the number of groups^9^.

Besides diagnosing the first cases, physicians from different backgrounds took part in the initiatives that resulted in the first measures for control of the disease. Ricardo Veronesi (professor at Faculdade de Medicina of the Universidade de Sao Paulo, founder of the Brazilian Society of Infectious Diseases, and responsible for administering interferon as a treatment for AIDS in Brazil),^n,o^gathered gay activists in March 1983, warning them about the need for a response from the government.

“[...] From then on, they started getting organized [...] to try to ensure some achievements, and, at the same time, discuss the AIDS issue and push authorities” (E8, 5/16/2011).

Motivated by the meeting and by the first AIDS cases in Brazil, Darcy Penteado scheduled an appointment with Sao Paulo’s Health Care Officer, João Yunes^15,24^.

“[...] a demand that had been started by people that had already [...] taken part in the gay rights movement. [...] and demanded the [State Health] Secretariat [...] take measures, provide guidance, give information regarding the epidemic [...]” (E26, 5/3/2011)

Former members of *Grupo Somos* and of the “*Lampião da Esquina*” newspaper, such as anthropologist Edward MacRae, Belgian filmmaker Jean Claude Bernadet, writer and journalist João Silvério Trevisan, and physician Valéria Petri (E8, E31), followed Darcy Penteado. They requested that Sao Paulo’s State Heath Secretariat (SES-SP) made a statement about AIDS and future cases treatment. This meeting motivated a movement for creating the AIDS Program at SES-SP, at Hansen’s Disease and Public Health Dermatology Division, which was coordinated by physician Paulo Roberto Teixeira, a former member of *Grupo Somos* (E26).

According to other studies^15,24^, the following contributed to the implementation of the AIDS Program at SES-SP: the first cases diagnosed; the demand for a group of militants, with support from a female physican; and the political conditions (democratic government, with sanitarians in strategic positions). The fear of the epidemic, the social group affected (an organized, upper class group of intellectuals with an important social capital), and the fact Sao Paulo had had a health care system with public health officer positions as of the 1960s should be added^p^, as well as the profile of the system’s director (a sanitarian and former member of the gay rights movement), a person with technical and political capability of dealing with stigmatized groups (E26).

Federal education and research institutions engaged by initiative of their professors and researchers. For example, Escola Paulista de Medicina, by physician Valéria Petri (E31), and Fiocruz, by its AIDS Research Laboratory. The latter was created in 1983, in Rio de Janeiro, by Bernardo Galvão, physician, master in Human Pathology, and PhD in Immunology, who became its coordinator, and Cláudio Ribeiro, a PhD in Immunohematology (E4).

The rise of Sao Paulo State AIDS Program confirmed the organization of a space to fight AIDS (AIDS Space). It was also a space for research and intervention, involving agents in the gay rights movement (militant space), in the medical field, teaching professionals, and researchers (scientific field), or staff in the state health care office (bureaucratic field).

### The Response from the Ministry of Health (1985-1989)

In February 1985, federal government initiatives for controlling AIDS were initiated through Professional Training for Controlling Hospital-Acquired Infections Program, which was coordinated by Luiz Carlos Pelizari Romero, from the National Secretariat for Special Health Care Programs (SNPES)^q^. Fabíola de Aguiar Nunes, a sanitarian physician, took part in the program as a representative from the Ministry of Education and Culture (E10).

When Carlos Sant’Anna, a physician, congressman, and one of the liaison agents in Tancredo Neves’ presidential election run, took over the Ministry of Health (MH) in March 1985, Fabíola Nunes took over SNPES, where, in the National Public Health Dermatology Division (DNDS), Brazil’s National Program on Sexually-Transmitted Diseases (STD) and AIDS (PNAids) would be created (E10, E11, E24).

The first measures taken by the MH were epidemiological surveillance, public health education and information, voluntary testing, and characterization of an epidemic that affected the whole population, to reduce stigma and discrimination of the most affected groups (E10, E11, E24).

Several social movements (homosexual, thalassemic, and hemophiliac) demanded a response from the government. The press insisted on the issue (E10, E26). Sao Paulo, Rio de Janeiro, and Rio Grande do Sul had already structured measures (E24), pressing the federal government to make a decision. The social representation of the disease, coupled with terror and fear, should be added to this.

In March 1985, meetings were held in Sao Paulo and Brasilia, to review and discuss the cases diagnosed. These meetings respectively resulted in the standardization of AIDS management procedures and the drafting of an ordinance by a panel of experts^r^. Epidemiological, clinical, and laboratory investigation were proposed, as well as public health education for suspected and confirmed cases, people in contact, and high-risk groups. The ordinance mentioned using condoms, disposable or individual syringes and needles, and controlling blood quality as preventive measures^s^. A training system was organized in Sao Paulo, for the states reporting their first cases (E10).

In January 1986, Fabíola Nunes invited dermatologist Maria Leide de Oliveira to DNDS (E10, E24). Miriam Franchini was responsible for STDs and sanitarian Lúcia Amaral, for AIDS, organizing an initial reporting system^19^. Lair Guerra de Macedo Rodrigues, a biomedical and professor at Universidade de Brasilia who had taken a specialization course at the Centers for Disease Control and Prevention (CDC) in the USA, was invited by Maria Leide to take over the AIDS program. The motivations behind this invitation were her experience at the CDC, her good references, and her potential influence and international support, as Carlyle Guerra de Macedo’s, a representative from the Pan American Health Organization (PAHO) was her brother (E24). As Maria Leide joined, a committee on risk group ^19^ was created. It was the precursor of Brazil’s National Aids Commission (CNAids) (E24) and it played an important technical and political role, by providing guidance to PNAids on the definition of adopted strategies^t^.

The initiatives aimed at structuring the program and preventing AIDS. The implementation of activities started being centrally managed by the MH, in a way to invert the relationship with the states, which by then created and executed their specific programs^24^.

AIDS was included in Brazil’s 8th National Health Conference, within “Epidemiological Surveillance” sub-topic. Later, the MH coordinated debates on “AIDS and Constitutional Convention” topic in the states, and this subsidized the national 1987 campaign, which discussed how the new health care system could solve the AIDS issue^u,v,w^.

The analysis of documents and interviews showed that Lair Guerra’s administration was marked by NGO criticism related to the care of patients, to the campaigns, and to the official discourse, which was considered normative and medicalizing. Criticism was mainly given against 1988’s campaign, which held individuals accountable for measures they were not capable of taking, such as controlling their blood quality, in a way to shift responsibility from the state to the people. Nonetheless, there were also people supporting the government campaign (Table 5).

**Table 5:** Some characteristics from the sub-spaces according to interviewed subjects and document analysis.

Sub-space	Characteristic
Militant space	Detachment of NGO/AIDS from the gay rights movement [...] we founded the Bahia Anti-AIDs Center, [...] to give lectures [...] at schools, universities, syndicates, neighborhood associations, the phrase gay group still caused prejudice, [...] it was a way to diversify and camouflage our actions [...]” (E20 on 3/23/2011, Salvador, BA). “[...] We just try to make it clear this is an anti-AIDS movement, not a gay movement[...]” (E3, on 6/20/2011, Sao Paulo, SP).
	Funding “At the time the first here was a fund from INPS, Hésio Cordeiro, [...] destined a share of the funds there to ABIA. [...]” (E29, on 6/15/2011, Rio de Janeiro, RJ). “[...] We set a space aside, as well as rooms and communication equipment: mail, telephone, etc., and this was the first, let us say, support. [...] Gapa was created inside the institution [SES-SP] [...]” (E26, 5/3/2011, Sao Paulo, SP).
	Main disputes “Gapa’s perspective leaned towards advocacy, ABIA had a concentration, a very high expertise to give information and guidance to specific groups” (E29, on 6/15/2011, Rio de Janeiro, RJ). “[...] the king ONGs were Gapa, *Pela Vida*, ABIA, and the others were all small” (E26, 5/3/2011, Sao Paulo, SP). “[...] The MSM category does not help anybody, nor does the prevention for bisexual individuals, not even to the queers and transvestites themselves, who often do not even consider they are men, then we protest against it, and still nowadays there are people defending it, but it is a misguided way as shown by the experience” (E20, on 3/23/2011, Salvador, BA).
	Conception regarding government campaigns [...] there is no real care program to help the sick people. [...] There is no consistent program for education or information. [...] In this campaign, the only information that is really clear is that the government does not know this country, nor the responsibilities it has to it [...]” ABIA Bulletin, no. 2, April 1988). “[...] even though it is late, shy, and filled with gaps, the government campaign has achieved tangible results in making different social layers aware [...]” (Luiz Mott, GGB, Letter sent to ABIA, ABIA Bulletin, no. 3, July 1988).
Scientific field	“After the Ministry of Health sent me an official passport, [...] so then I could represent Brazil in the United States, they started contradicting me [...]” (E31, 6/20/2011, Sao Paulo, SP). “They did not accept that other agency instead of Fiocruz would do it. [...] I managed to get grants in the United States. UFRJ had an agreement signed with Fiocruz. [...] The Ministry of Health did not give statements approving the project. [...] However, as they were not the ones that would do it, they canceled it. Two and a half million dollars” (E7, 6/15/2011, 12/7/2011, and 12/8/2011, Rio de Janeiro, RJ, Skype).
Medical field	“[...] At that time, they thought a dermatologist could disturb their work or that which they intended to do. So they said that on television. Once Veronesi said “A dermatologist only serves to disrupt” [...] (E31, 6/20/2011, Sao Paulo, SP).
Bureaucratic field	“[...] all the programs, at a certain point, were organized in public health dermatology offices in the states. Similar to what had happened in Sao Paulo and because of the relationships we had. [...] at that time, the whole public health dermatology department of the Ministry of Health, in 1983, 1984, specially, had a very indecisive stance, even opposing us. [...] that caused a certain agitation in the ministry concerning the topic [...]” (E26, 5/3/2011, Sao Paulo, SP).

NGO/AIDS: Specific AIDS-related non-government organization; INPS: National Institute of Social Security; ABIA: Brazilian Interdisciplinary AIDS Association; Gapa: Aids Support and Prevention Group; SES-SP Sao Paulo’s State Health Secretariat; MSM: Men who have sex with men; GGB: Bahia’s Gay Group; FIOCRUZ: Oswaldo Cruz Foundation; UFRJ: Universidade Federal do Rio de Janeiro

The relationship between the disease, sexuality, and use of injection drugs led its concept to be influenced by the religious discourse. Catholic tradition especially instilled the impression the disease was related to sin, moral transgression, and divine punishment, opposite to the discourse from homosexual groups^x^. This issue was mentioned by some subjects interviewed, and it shows the importance of analyzing the roles of agents in this field in setting the policy.

When it started being referred to as Acquired Immunodeficiency Syndrome (AIDS) (*Síndrome da Imunodeficiência Adquirida –* SIDA, in Portuguese), the foreign term was incorporated by physicians and the press in Brazil. Was the incorporation of a foreign word submission from scientific and cultural field agents to the USA, or simply a wish for better international communication in the scientific field? When asked, one of the interviewed subjects shows that, in the MH, its adoption was rationalized and formalized in a meeting for defining the term to be used.

“[...] I was present in the main meeting when they decided Brazil was not going to refer to it as SIDA [...] to keep from stigmatizing all Cidas [short for Aparecida, a common female name in Brazil]. That is why AIDS was chosen” (E11, 8/4/2011).

The associations that fight AIDS (NGO/AIDS), which rose from 1985, had a determining role for regulating the control of blood and blood products^23^ and in the lobby in the constitutional convention for pushing the blood issue^20,y^.

The empirical material showed that the main disputes between agents in the PNAids and in the association movement revolved around the preventive campaigns (target population, technical or popular language, what could be said, which terms to use, among other things). And from the release of azithromycin (AZT), also regarding treatment.

During the whole period analyzed, the technical conception from the bureaucratic field prevailed in the official discourse. However, due to the influence from militant and religious fields, AIDS was considered to be a threat to society in general rather than restricted to specific groups.

Lair’s international negotiations with the CDC and PAHO, as well as her management ability, contributed to the initial structuring and extension of PNAids. The Program also had important support from the Special Public Health Care Services Foundation for its interiorization, through its representative for transmittable diseases, sanitarian physician Pedro Chequer, who later was part of PNAids team^19^.

Getting PNAids attached to DNDS was under dispute, and this resulted from disagreements between agents from the medical field (Fabíola and Maria Leide) and AIDS Space, which was consolidating itself and seeking autonomy (Lair Guerra) (E10). In 1987, after Fabíola Nunes left SNPES, Brazil’s National STD/AIDS Division^19^ was created. It was no longer part of DNDS and had autonomy regarding public health dermatology, a medical specialty. Its creation and transference to Brazil’s National Office of Basic Health Care Initiatives were made official in 1988. Lair remained in the management of the division until March 1990, during the early Collor administration.

### AIDS Space: Its Agents and Sub-spaces

The following people affected by the epidemic engaged in AIDS Space: HIV positive people, people who were more vulnerable to AIDS (homosexuals, injection drug users, people with hemophilia, sex workers); their parents and friends; professionals related to the epidemic, especially physicians and researchers. Those who enter this space for personal reasons, in general, joined the militant sub-space, had diverse educational backgrounds, and important cultural capital, with master’s or PhD’s degrees. In the bureaucratic and scientific sub-spaces, physicians predominated; the scientific field agents had graduate diplomas or were attending such courses (Table 3).

Despite the importance of the gay movement in planning the first responses to the epidemic, the NGO/AIDS were created seeking get detached from this movement (E3, E20) (Table 5).

The first NGO/AIDS were funded by the State: Aids Support and Prevention Group (Gapa) by SES-SP (E3, E26), and Brazilian Interdisciplinary AIDS Association (ABIA) by the National Social Security Institute of Medical Care (E29) and by Finep^20^, through public health militants taking over positions in these institutions, such as Hésio Cordeiro and Reinaldo Guimarães.

In this sub-space, the main issues were funding, structure, and liaison with the PNAids, living with HIV/AIDS, strategies for prevention and use of the technical formulation “men who have sex with men” (MSM). Disputes were also between the NGO/AIDS and groups in which AIDS was a cross-sectional topic, NGOs with more political perspectives (ABIA) and NGO focusing on care (Gapa) (E3, E12, E14, E18, E20, E29, E33) (Table 5).

In the scientific field, young researchers and new PhD engaged themselves, seeking independent and original research lines. The main disputes were between generations or institutions. What was at stake was the recognition of the scientific authority or the position of state expert (E4, E7, E31) (Table 5).

In the medical field, the disputes were between dermatologists, infectious disease specialists, tropical medicine, and infectious and parasitic diseases (E4, E31) (Table 5).

In the bureaucratic field, the disputes were between State Health Secretariats and PNAids (E10, E26) (Table 5). Some agents had priority insertion in this field, where agents from the remaining sub-spaces circulated through the CNAids or by taking over positions at PNAids or the remaining management levels.

The meeting of conceptions from the different sub-spaces on AIDS contributed to a broad construction of the problem and actions, based on the dignity of patients and human rights. CNAids, as the State commission that concentrated scientific and militant powers, transmuted into bureaucratic power, had an important role in planning the official discourse, which enabled negotiations between agents from different sub-spaces.

### Political and Militant Path

Some of the agents involved in the genesis of the policy had a dominant political path, by occupying elective offices (Carlos Sant’Anna and Sérgio Arouca). Others had taken part in the sanitary movement (E9, E10, E26, E27), in the gay rights movement (E8, E18, E20, E26, E33), and in the fight against dictatorship, in clandestine parties such as Brazil’s Communist Party (E4, E12), and *Ação Popular* (Popular Action) Party (E26) Most agents who had political capital to some extent stood out in the militant space, taking over dominant positions, such as the management of NGO/AIDS or other associative organizations (Table 3).

### Final Considerations

This socio-historical analysis allowed explaining how the initial demand from militants to SES-SP took place, highlighting the role of the scientific field, from the start of the epidemic. It also recovered the initial formulation of the policy at the national level, which had rarely been described in the literature, identifying the main agents involved and showing the implementation of initiatives already in the last months of the military government. It also showed the importance of the participation of sanitarians who took over government positions at that time.

AIDS Space was historically constituted as a space for fighting for the organization of the response to the epidemic and public health care intervention. The authority to talk about the meaning of the disease, the methods to prevent, and treat it were under dispute, as well as strategies for controlling the epidemic. Its structure involved agents from the medical field, from “Collective Health Space”, from the bureaucratic field, from the gay rights movement, and from the scientific field, influenced by political, religious, and juridical fields. Later, movements of people with hemophilia, people with thalassemia and sex workers, and NGO/AIDS, composing the militant sub-space.

The government response had the level of government that implemented the first initiatives as a specific aspect. Traditionally, the MH had a national policy, which was followed and enforced by the states. The initiatives against AIDS were started in the states, when MH denied the need for intervention. The national policy was implemented since 1985. The conditions that enabled including AIDS in the political agenda were the following: the democratic transition process; sanitarians taking over management positions at the MH; the quick spread of the epidemic; the existence of research groups of infectious and parasitic diseases, involving pathologists who became immunologists; the advancements in the medical field regarding the disease, and the development stage of the clinical and laboratory research; the organization of AIDS programs by some states; and the pressure from social movements and the press.

The comprehension of the historical reasons that allowed the initial planning of this policy and the analysis of the paths of agents and struggles common to AIDS Space and to the “Collective Health Space” empirically show the relationship between the policy and the sanitary reform movement and its assumptions. Its origin, during a time when the sanitary reform movement was rising and when there was no specific therapy, contributed to the initial prioritization of prevention. The dominance of the medical field and the constitution of the Brazilian Unified Health System (SUS) contributed to a connection between preventive measures and ensured treatment, a component that is necessary for implementing a policy that is based on integral care, which is internationally considered as an example.

## Footnotes

a Guerra MAT. Política de controle da AIDS da Secretaria de Estado da Saúde de São Paulo, no período 1983-1992: a história contada por seus agentes [dissertation]. São Paulo (SP): Faculdade de Medicina da Universidade de São Paulo; 1993.

b França MSJ. Ciências em tempos de AIDS: uma análise da resposta pioneira de São Paulo à epidemia [these]. São Paulo (SP): Pontifícia Universidade Católica de São Paulo; 2008.

c Santos VL. Avaliação do grau de implementação do Programa de DST/AIDS no Distrito Sanitário Especial Indígena de Mato Grosso do Sul – Distrito de Dourados [dissertation]. Rio de Janeiro (RJ): Escola Nacional de Saúde Pública da Fundação Oswaldo Cruz; 2009.

d Lima SCVS. Avaliação do processo de implementação da política de controle das DST/AIDS no Maranhão [these]. São Luís (MA): Universidade Federal do Maranhão; 2006.

e Monteiro ALO. A relação Estado e Sociedade Civil no processo de formulação e implementação de políticas públicas: análise do Programa Nacional de DST e AIDS (1980-2006) [dissertation]. Brasília (DF): Faculdade de Economia e Administração da Universidade de Brasília; 2006.

f Campos LCM. Estado e terceiro setor na prestação de serviços públicos: o Programa Nacional de DST/AIDS: 1994-2005 [dissertation]. São Paulo (SP): Escola de Administração de Empresas de São Paulo da Fundação Getúlio Vargas; 2005.

g Silva CLC. Ativismo, ajuda mútua e assistência: a atuação das organizações não-governamentais na luta contra a aids [these]. Rio de Janeiro (RJ): Universidade Federal do Rio de Janeiro; 1999.

h Barboza R. Gestão do Programa Estadual DST/AIDS de São Paulo: uma análise do processo de descentralização das ações no período de 1994 a 2003 [dissertation]. São Paulo (SP): Coordenadoria de Controle de Doenças da Secretaria de Estado da Saúde de São Paulo; 2006.

i Stanise VL. Avaliação do grau de institucionalização dos programas municipais de DST/HIV/AIDS na Regional de Saúde de Campinas, São Paulo, Brasil [dissertation]. Rio de Janeiro (RJ): Escola Nacional de Saúde Pública da Fundação Oswaldo Cruz; 2008.

j Barros SG. A política nacional de luta contra a aids e o espaço aids no Brasil [these]. Salvador (BA): Instituto de Saúde Coletiva da Universidade Federal da Bahia; 2013.

k Collective Health, in its genesis, according to the findings from an empirical study conducted by Vieira-da-Silva and Pinell (2013), analyzing the paths of 26 founders, could be interpreted as a social space that aimed to become a field but still did not have relative autonomy or a common *habitus*, found in consolidated fields, such as scientific, medical, bureaucratic, and political fields.

l Silva LL. Homossexualidade e aids em São Paulo [dissertation]. São Paulo (SP): Pontifícia Universidade Católica de São Paulo; 1986.

m Silva LL. Homossexualidade e aids em São Paulo [dissertation]. São Paulo (SP): Pontifícia Universidade Católica de São Paulo; 1986.

n Interferon teve seus poderes superestimados. *Folha de São Paulo*. 4 jun 1983.

o Congresso termina com crítica de médico ao Inamps. *Folha de São Paulo*. 6 jun 1983.

p Ribeiro ES. Gestão estadual do SUS: alternativas e adequação do sistema de informação para a política hospitalar [dissertation]. São Paulo (SP): Faculdade de Ciências Médicas da Santa Casa de São Paulo; 2008.

q Infecção hospitalar. *Folha de São Paulo*. 17 dez 1985; Cidades:18. Pastoral pede clareza na campanha. *Folha de São Paulo*. 26 fev 1987.

r Histórico da aids: documento do Ministério da Saúde, SNEPS/DNS. Dr. Fabíola Nunes’ Personal File.

s Ministério da Saúde. Portaria nº 236 de 2 de maio de 1985. Define critérios diagnósticos e estratégias de ação para o controle da epidemia da aids. *Diario Oficial Uniao*. 6 mai 1985*.*Available from: http://sna.saude.gov.br/legisla/legisla/aids/GM_P236_85aids.doc

t Ministério da Saúde, Secretaria de Políticas de Saúde, Coordenação Nacional de DST e AIDS. Comissão Nacional de AIDS: 1986-1994. Brasília (DF); 1994.

u Médico diz que detentos internados não apresentam sintomas de aids. *Folha de São Paulo*. 8 out 1986.

v Ministério da Saúde. AIDS e Constituinte: subtema da VIII Conferência Nacional de Saúde. Brasília (DF); 1986.

w Teixeira PR, Oliveira MLWDR, Pluciennik TI, Rosenthal C, Petri V, Koperstych S, et al. Debate AIDS. Interviewer: Rodolfo Gamberini. São Paulo: TV Cultura; 1987. Transcribed interview given to *Roda Viva* TV show on January 19, 1987. Available from: http://www.rodaviva.fapesp.br/materia/211/entrevistados/debate_aids_1987.htm

x Pastoral pede clareza na campanha. *Folha de São Paulo*. 26 fev 1987.

y Contrera WF. GAPAs: uma resposta comunitária à epidemia da AIDS no Brasil. Brasília (DF): Ministério da Saúde; 2000.
