# Erratum

**DOI:** 10.1590/S1518-8787.2016050005801err

**Published:** 2016-11-29

In the Portuguese version of the article: **“The genesis of the AIDS policy and AIDS Space in Brazil (1981-1989)”** published in the “Revista de Saúde Pública”, volume 50 (2016), number 43, DOI:10.1590/S1518-8787.2016050005801, the following references should be added at the end of section **References**: 

24. Teixeira PR. Políticas públicas em AIDS. In: Parker R, organizador. Políticas, instituições e AIDS: enfrentando uma epidemia no Brasil. Rio de Janeiro (RJ): Jorge Zahar; 1997. p.43-68.

25. Vieira-da-Silva L, Pinell P. The genesis of collective health in Brazil. Sociol Health Illn. 2014;36(3):432-46. DOI:10.1111/1467-9566.12069

