# Erratum

**DOI:** 10.11606/S1518-8787.2018052005801err

**Published:** 2018-02-27

In the article: “**The genesis of the AIDS policy and AIDS Space in Brazil (1981-1989)**” published in the “Revista de Saúde Pública”, 2016;50:43, DOI: http://dx.doi.org/10.1590/s1518-8787.2016050005801; on page 8, 7th paragraph.

**Where it reads:**

“azithromycin (AZT)”

**It should read:**

“zidovudine (AZT)”.

